# Number 2 Feibi Recipe Ameliorates Pulmonary Fibrosis by Inducing Autophagy Through the GSK-3β/mTOR Pathway

**DOI:** 10.3389/fphar.2022.921209

**Published:** 2022-07-12

**Authors:** Haoge Liu, Qinglu Pang, Fang Cao, Zhaoheng Liu, Wan Wei, Zhipeng Li, Qi Long, Yang Jiao

**Affiliations:** ^1^ Graduate School, Beijing University of Chinese Medicine, Beijing, China; ^2^ Dongfang Hospital Affiliated to Beijing University of Chinese Medicine, Beijing, China; ^3^ Department of Respiratory and Critical Care Medicine, Chongqing Traditional Chinese Medicine Hospital, Chongqing, China

**Keywords:** number 2 Feibi Recipe, idiopathic pulmonary fibrosis, oxidative stress, autophagy, Chinese medicine

## Abstract

Number 2 Feibi Recipe (N2FBR) is a traditional Chinese medicine formula for treating idiopathic pulmonary fibrosis. N2FBR inhibits H_2_O_2_-mediated oxidative stress damage in alveolar epithelial cells by increasing autophagy, as we previously demonstrated. However, it is unknown if similar mechanisms occur *in vivo*. We established a pulmonary fibrosis model by instilling bleomycin (BLM) from the airway to examine the effects of N2FBR on pulmonary fibrosis and investigate its probable mechanism in this work. We discovered that N2FBR treatment effectively alleviated interstitial fibrosis as well as collagen deposition, primarily in upregulating SOD, GSH-Px, T-AOC and downregulating MDA content. N2FBR also increased the expression of LC3B, Beclin-1, LAMP1, TFEB and downregulated the expression of p62, legumain. N2FBR treatment boosted the production of autophagosomes, according to the results of the TEM observation. Furthermore, we explored that N2FBR exerted its anti-oxidative stress and pro-autophagy effects via GSK-3β/mTOR signalling pathway. Therefore, these results provide further evidence for the protective effect of N2FBR in pulmonary fibrosis. Our findings could have ramifications for the development of antifibrosis therapies.

## Introduction

Idiopathic pulmonary fibrosis (IPF) is a progressive interstitial pneumonia illness that has no known cause, and has a median survival duration of 3–5 years after diagnosis (Lederer and Martinez, 2018). Histologically, the disease is characterized by significant destruction of the alveolar structure as well as extracellular matrix (ECM) deposition (Raghu and Richeldi, 2017). IPF patients have a median age of approximately 65 years when they are diagnosed. It is more prevalent in ex-smokers and results in dyspnea, dry cough, and progressive loss of respiratory function (Glassberg, 2019). In the last few years, several new antifibrotic agents such as pirfenidone (King et al., 2014) and nintedanib (Nasser et al., 2021) have been introduced. These medications slowed the deterioration of lung function in IPF patients but had no effect on survival or quality of life. Lung transplantation remains the only treatment that enhances patient survival. It is crucial to have a deeper understanding of the cellular processes and molecular pathways involved in order to develop effective treatments.

In comparison to other organs, the lungs are especially vulnerable to oxidate stress. Accumulating to mounting evidence, oxidative stress plays a key role in the initiation and progression of IPF. Apoptosis of alveolar epithelial cells (AEC) and apoptosis resistance in myofibroblasts are caused by excessive production of reactive oxygen species (ROS) by defective mitochondria, resulting in enhanced collagen deposition and fibrotic foci development (Tian et al., 2019). Autophagy is a process in which damaged organelles or proteins are degreed by lysosomes as a possible survival mechanism amid oxidate stress (Racanelli et al., 2018). The accumulation of p62 and low levels of LC3-II were detected in lung tissue samples from IPF patients (Patel et al., 2012). The mammalian target of rapamycin (mTOR) is an autophagy-related regulating protein kinase. Glycogen synthase kinase 3β (GSK-3β) is a serine/threonine kinase with many functions. mTOR is the downstream target of GSK-3β, according to numerous studies. SB216763 is a GSK-3β inhibitor. Simultaneously, previous research suggested that SB216763 alleviated pulmonary fibrosis by inhibiting inflammatory cytokines (Gurrieri et al., 2010) and regulating TGF-β1-induced myofibroblast differentiation (Baarsma et al., 2013). These findings proposes that autophagy plays a role in the molecular pathways that lead to the IPF development. Nevertheless, previous research on pulmonary fibrosis mainly focuses on the formation of autophagosomes. The autophagosome and lysosome fusion is significant for complete autophagic flux. Oxidative stress damages the membrane permeabilization of lysosomes, which result into lysosome dysfunction (Qin et al., 2017). Lysosomal defects disrupt the fusion of autophagosomes and lysosomes, therefore, inhibiting autophagic flux (Yu et al., 2018). The relationship between oxidative stress and autophagy in pulmonary fibrosis requires further investigation.

Number 2 Feibi Recipe (N2FBR), a Chinese medicine herbal formula, is used to treat respiratory disorders such as idiopathic pulmonary fibrosis. It is a modified formula from Feibi Recipe (FBR). FBR is comprised of eight herbs: Sheng Huangqi (Astragalus membranaceus Bge), Jin Yin Hua (Flos Lonicerae), Sheng Gan Cao (Radix Glycyrrhizae), Danggui (Angelicae Sinensis Radix), Chuan Shan Long (Discorea nipponica Makino), Zhe Bei Mu (Bulbus Fritillariae Thunbergii), and Shiwei (Pyrrosia lingua (Thunb.) Farwell), and Gua Lou Pi (Pericarpium Trichosanthes). The Feibi recipe inhibited bleomycin-induced pulmonary fibrosis through modulating TGF-β1/Smad3 Signalling Pathways (Wang et al., 2020). FBR-medicated serum crucially inhibited the production of pro-inflammatory cytokines induced by LPS in RAW264.7 macrophages *in vitro*. Additionally, treatment with FBR-medicated serum suppressed the activation of NF-κB and Smad2/Smad3 (Wei et al., 2022). N2FBR, an advanced version of FBR, comprises of the following commonly used Chinese herbs: Sheng Huangqi (Astragalus membranaceus Bge), Hong jingtian (herba rhodiolae), Jin Yin Hua (Flos Lonicerae), Huang Qin (Radix Scutellariae), Dan Shen (Radix Salviae Miltiorrhizae), and Sheng Gan Cao (Radix Glycyrrhizae). In a large body of clinical practise, N2FBR has been demonstrated to relieve symptoms and enhance the quality of life of IPF patients. N2FBR has been shown to have anti-oxidative effect of N2FBR both *in vitro* and *in vivo* (Liu et al., 2018; Gu et al., 2022). Moreover, N2FBR promoted autophagy in H_2_O_2_-mediated AECs. Nevertheless, the therapeutic mechanisms of N2FBR in pulmonary fibrosis are not fully understood. The goal of this study was to see if N2FBR could reduce oxidative stress in BLM-induced lung fibrosis by triggering autophagy via the GSK-3β/mTOR pathway.

## Materials and Methods

### Chemicals and Reagents

Number 2 Feibi Recipe (N2FBR) comprised of six herbs, including 30 g Sheng Huangqi (Astragalus membranaceus Bge), 30 g Hong jingtian (herba rhodiolae), 30 g Jin Yin Hua (Flos Lonicerae), 20 g Huang Qin (Radix Scutellariae), 20 g Dan Shen (Radix Salviae Miltiorrhizae), and 10 g Sheng Gan Cao (Radix Glycyrrhizae). All the herbs were purchased from Beijing Kang Ren Tang, a Pharmaceutical Industry Co. The herbs had initially been soaked in water for 30 min. Afterwards, they were boiled for 1 h. Following filtration, the liquid was boiled for 0.5 h, sealed, vacuum dried, as well as stored in a glass bottle at 4°C until use. SB216763 was purchased from MedChem Express (Shanghai, China) and dissolved in a vehicle (25% dimethyl sulfoxide, 25% polyethylene glycol, and 50% saline).

Bleomycin was provided by Fresenius Kabi (Lake Zurich, United States). The HE Stain and Masson’s Trichrome Stain Kit were provided by Solarbio (Beijing, China; G1120, G1340, respectively). Mice MDA, SOD, and T-AOC assay kits were purchased from Jiancheng Bioengineering Institute (Nanjing, China; A015-2-1, A003-1-2, A001-3-2, respectively). The TGF-β1 Elisa kit had been provided by Elabscience (Wuhan, China; E-EL-0162c). The rabbit anti-LC3A/B, rabbit anti-SQSTM1/p62, rabbit anti-Beclin 1, rabbit anti-LAMP1, rabbit anti-total-mTOR, rabbit anti-phospho-mTOR (Ser2448), rabbit anti-total-GSK3 beta, rabbit anti-phospho-GSK3 beta (Ser9), goat anti-rabbit IgG-HRP, as well as goat anti-rabbit IgG-FITC were purchased from Affinity (Nanjing, China; AF5402, AF5384, AF5182, DF7033, AF6308, AF3308, AF5016, AF 2016, S0001, S0008, respectively). Bioss provided the rabbit anti-Collagen alpha-1(I), rabbit anti-Legumain, and rabbit anti-TFEB antibodies (Beijing, China; bs-20124R, bs-3907R, bs-5137R, respectively).

### Animals

All mice experiments were approved by the Animal Ethical Experimentation Committee of Beijing University of Chinese Medicine and performed in accordance with the guidelines of the Institutional Animal Care and Use Committee. A total of 40 C57BL/6J male mice weighing 22–24 g were purchased from Beijing Vital River Labomouseory Animal Technology Co., Ltd. (Beijing, China). The mice were housed in standard laboratory conditions, with filtered air, ambient temperature (22°C–26°C), humidity (55 ± 10%), and light (12 h light/dark cycle). The mice were provided with sterilized water and food.

### Animal Model and Experimental Design

The mice were randomly assigned into four groups (*n* = 10 per group): a sham group (Sham), a model group (Model), number 2 FBR group (N2FBR), and SB216763 group (SB). As previously described (Tong et al., 2021), the mice were anesthetized with isoflurane solution, and then instilled with bleomycin solution via the airway (2 u/kg), except for mice in the sham group, which received the same volume of saline solution. The N2FBR group was given the number 2 Feibi Recipe (21.02 g/kg) by gavage once a day on the second day after modelling, whereas the other group received an equivalent quantity of saline. The daily dosage of N2FBR in mice was confirmed using the conversion ratio of the surface area between mice and humans. Additionally, SB group mice were administered with SB216763 (20 mg/kg) dissolved in vehicle (25% dimethyl sulfoxide, 25% polyethylene glycol, and 50% saline) (Gurrieri et al., 2010) twice a week by intraperitoneal injection, while other group received equal volumes of vehicle intraperitoneally. Bodyweight had been measured every 3 days, and the mice were euthanized after 21 days.

### Hematoxylin-Eosin and Masson Staining

The left lung was promptly fixed in 4% paraformaldehyde and embedded in paraffin. Tissue slices with a thickness of 3-μM-thick tissue sections. Histological changes as well as collagen deposition were observed using hematoxylin-eosin (H&E) staining and Masson trichrome staining. HE staining and Masson staining was performed, while dewaxing and hydration had been conducted according to the manufacturer’s instructions. Alveolitis and Ashcroft scores were used to provide semi-quantitative study of inflammation. The area positive for Masson trichrome had been measured, and the ratio of the Masson trichrome-positive area to the total fibrotic area was calculated using ImageJ software.

### Immunohistochemical and Immunofluorescence Staining

Tissue sections were dewaxed with xylene and rehydrated as per standard protocols. Antigen retrieval was done in a microwave oven by boiling potions in 10 mM citrate buffer (pH6). After that, sections were immersed in 3%H2O2 for 20 min and blocked with 10% goat serum. The primary antibodies against Collagen I, P62, and Legumain were then added and incubated at 4°C overnight. The following day, slides were incubated with secondary antibodies as well as stained with a DAB reagent. A light microscope was used to photograph all of the slices.

The dewaxing, rehydration, and antigen retrieval methods for immunofluorescence staining were comparable to those employed for IHC. After blocking, the sections were incubated overnight with the primary antibody against TFEB in 5% BSA/0.1% Triton X-100 PBS. The slides were stained with FITC-conjugated secondary antibody and DAPI. Images had been quantified using ImageJ software.

### Determination of Oxidative Stress Biomarkers and Fibrosis Biomarkers

The contents of malondialdehyde (MDA), superoxide dismutase (SOD), glutathione peroxidase (GSH-Px), and total antioxidant capacity (T-AOC) in serum was determined using commercially available MDA, SOD, GSH-Px, and T-AOC kits. The levels of hydroxyproline (HYP), collagen III (Col III), and α-smooth muscle actin (α-SMA) in lung tissues were determined using a commercially available kit according to the manufacturer’s protocol.

### Western Blot

RIPA lysis buffer was used to extract total protein from lung tissues, which was then centrifugation for 15 min at 12,000 rpm at 4°C. A BCA protein concentration kit was used to determine the protein (MDL, cat. no. MD913053) concentration. Each well had been loaded with equal amounts of protein as well as separated using 10% SDS PAGE. Proteins were transferred to 0.22 µM PVDF membranes (Millipore, United States), which were blocked with 5% nonfat milk for 2 h. Membranes were incubated with specific primary antibodies overnight at 4°C. Membranes were treated with secondary antibodies conjugated with HRP for 1 h at room temperature after primary incubation. ECL reagent was used to visualise the signals. All determinations had been performed independently and repeated three times.

### Transmission Electron Microscopy

The autophagy-related structure was seen using transmission electron microscopy (TEM). Lung tissues were fixed in 2.5% glutaraldehyde and post-fixed in 1% osmium tetroxide for 2 h. Dehydration was performed using a graded ethanol series. The samples were embedded in epoxy and heat-cured overnight and sliced into 90 nm-thick pieces. The sections were viewed under TEM after being stained with uranyl acetate and lead citrate (JEM-1400, JEOL, Tokyo, Japan).

### Statistical Analysis

GraphPad Prism 8 was used to conduct the statistical analysis (GraphPad Software, Inc.). Each experiment was repeated at least 3 times. All results had been expressed as mean ± standard deviation (SD). One-way ANOVA or Two-way ANONA followed by Tukey’s test were used for comparison between multiple groups. Brown-Forsythe test followed by the Welch ANOVA test was used for determining the Ashcroft score and inflammation. *p* < 0.05 was considered statistically significant.

## Results

### Deaths and Body Weight Changes

**FIGURE 1:**
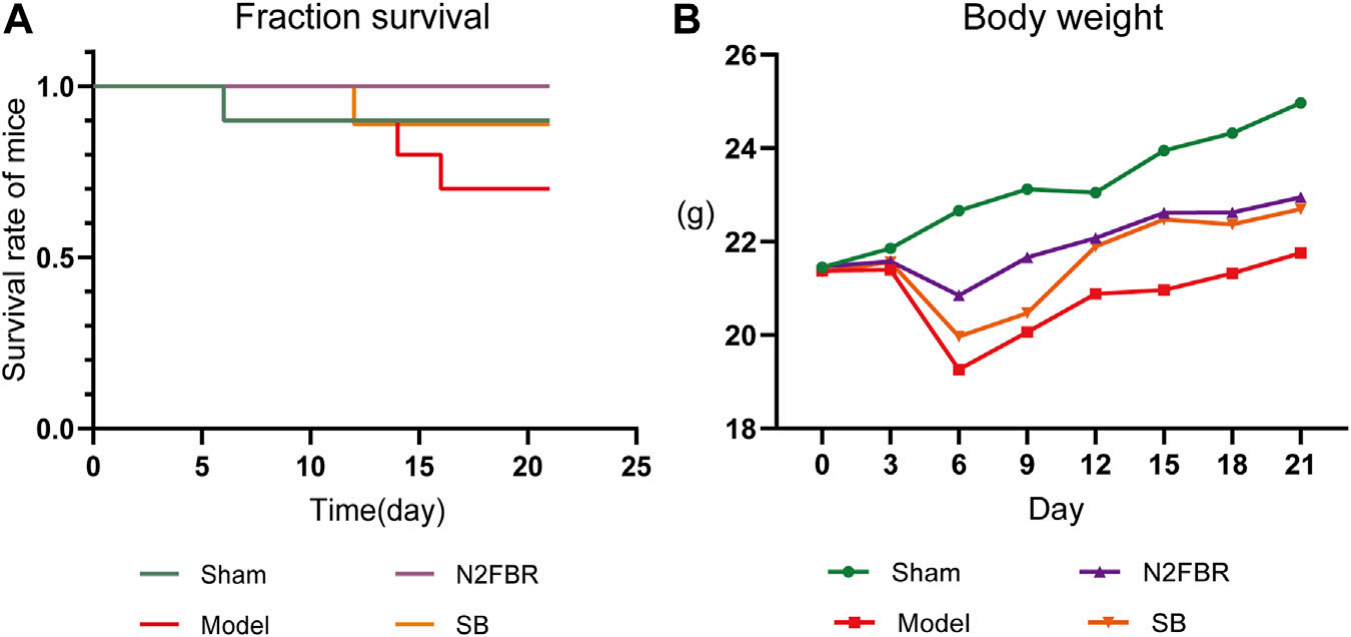
Number of deaths and changes in body weight of mice (*n* = 10 mice/group). **(A)** The survival curve of mice. **(B)** Changes in body weight of mice.

As shown in Figure 1A, one mouse in the sham group died on day 6, while a mouse in the model group died on days 6, 14, and 16. On day 12, one mouse died in the SB group. There were no deaths in the N2FBR group. There were 1, 3, and 1 death in the sham group, model group, and SB group, respectively. As illustrated in Figure 1B, with the exception of the Sham group, the weights of the mice were crucially reduced on day 6 after model construction. The weight of mice, on the other hand, continued to recover 9 days after the model was built. In comparison to the Sham group, the final mice weights in the model group were significantly reduced (*p* < 0.01). Although the body weights of mice in the N2FBR and SB groups increased, no statistically significant difference was observed when made comparison with the model group.

### Effects of Number 2 Feibi Recipe on the Histopathology of Bleomycin-Induced Pulmonary Fibrosis

**FIGURE 2:**
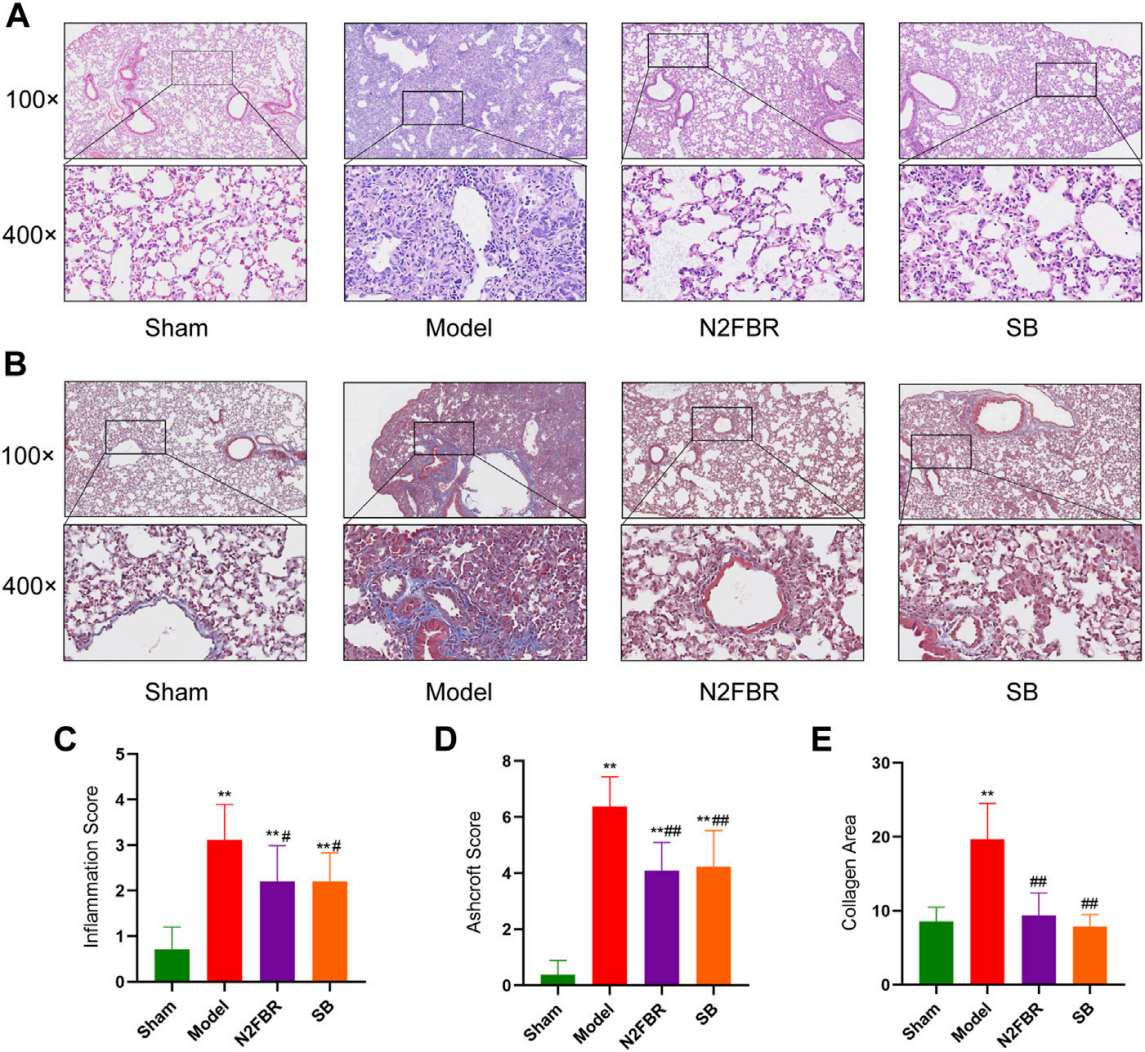
N2FRB alleviated BLM-induced pulmonary fibrosis. **(A–B)** Representative images of HE staining, MASSON staining. **(C–E)** Quantitative analysis of Alveolitis score and Ashcroft score based on HE staining and collagen area based on MASSON staining assays. Data were analyzed using one way ANOVA (Mean ± SD): **p < 0.01 compared with the sham group; #*p* < 0.05, ##*p* < 0.01, compared with the BLM group.

To determine the degree of lung injury and fibrosis following treatment, sections of lung tissue were stained with H&E and Masson trichrome. Based on HE staining (Figure 2A), the structure of the pulmonary alveoli in the model group was severely damaged. The pulmonary alveolar walls had been thickened. A large amount of inflammatory cell infiltration was observed. Nevertheless, N2FBR and SB significantly ameliorated the effects of BLM. Meanwhile, therapy with N2FBR (*p* < 0.05, *p* < 0.01) and SB (*p* < 0.05, *p* < 0.01) significantly lowered the alveolitis score (Figure 2C) and Ashcroft score (Figure 2D), which indicate the severity of inflammation and fibrosis, respectively. The sham group’s pulmonary alveoli tissues had a normal structure, with only light blue fibers observed. Unsurprisingly, large quantities of blue collagen had been deposited in the model group. Blue collagen deposition was significantly alleviated in the N2FBR (*p* < 0.01) and SB(*p*<0.01) groups in contrast to the model group (Figures 2B,E). Taken together, treatment with N2FBR significantly reduced bleomycin-induced pulmonary fibrosis.

### Number 2 Feibi Recipe Regulates the Expression of Alpha Smooth Muscle Actin, Collagen- I, Collagen-III, Hydroxyproline, and TGF-β1

**FIGURE 3:**
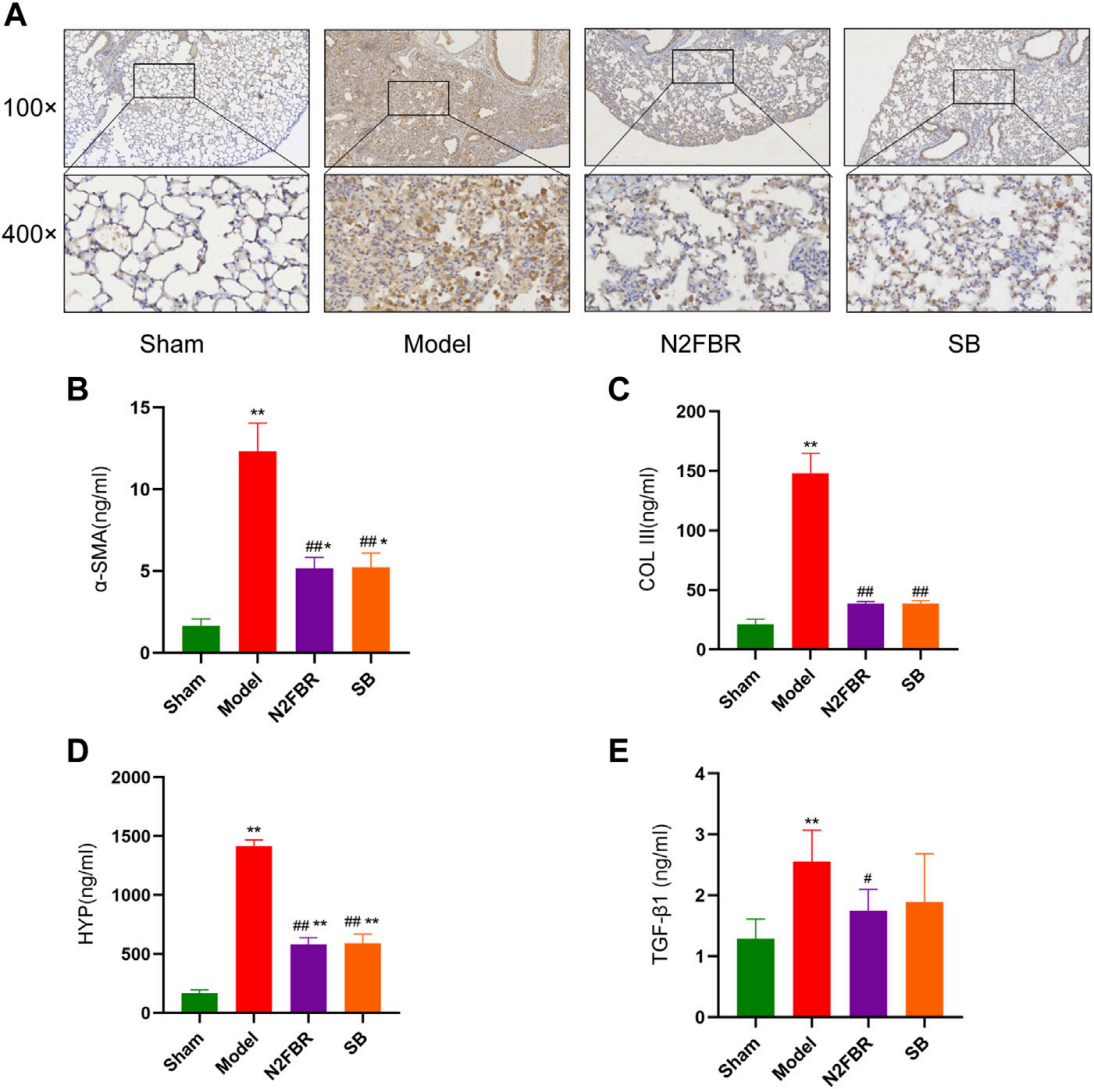
Effects of N2FBR on fibrosis related markers. **(A)** Immunohistochemistry assays for Col I in different groups. **(B–E)** Total serum levels of α-SMA, Col III, HYP and TGF-β1. Data are expressed as Mean ± SD: **p < 0.01, compared with sham group, #*p* < 0.05, ##*p* < 0.01, compared with model group.

The extracellular matrix’s principal collagen proteins are collagen-I, collagen-III, and collagen-SMA. The immunohistochemical staining of collagen I revealed higher expression of collagen-I in the model group, which had been attenuated by N2FBR and SB treatment (Figure 3A). HYP levels are indicative of tissue collagen content. α-SMA, Collagen-III, and HYP were measured by ELISA. N2FBR decreased the expression levels of α-SMA, Collagen-III, and HYP in bleomycin-induced lung tissue (Figures 3B–D). TGF-β1 plays a crucial part in tissue fibrosis, by stimulating fibroblast proliferation and collagen synthesis (Liu et al., 2016). Compared to the model group, the TGF-β1 contents had been significantly decreased (*p* < 0.05) in the N2FBR group (Figure 3E). Nevertheless, there was no statistically significant difference between the SB and model groups. It has been indicated by the findings that N2FBR is effective against the progression of lung fibrosis by reducing collagen deposition.

### Number 2 Feibi Recipe Alleviates Oxidative Stress in Bleomycin-Induced Pulmonary Fibrosis Mice

**FIGURE 4:**
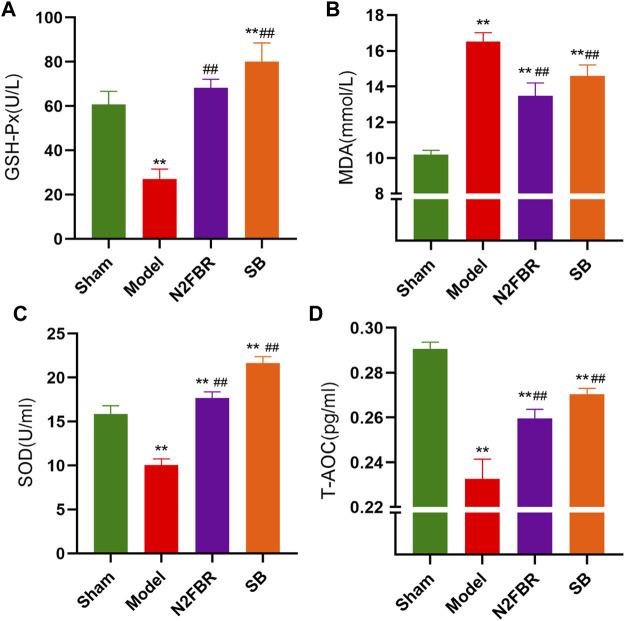
Effects of N2FBR on oxidative stress. Total serum levels of **(A)** GSH-Px, **(B)** MDA, **(C)** SOD, **(D)** T-AOC as measured through ELISA. Data are expressed as Mean ± SD: **p < 0.01, compared with sham group, ##*p* < 0.01, compared with model group.

To see if N2FBR contributes to anti-oxidative stress, researchers measures the activities of MDA, SOD, T-AOC, and GSH-Px. GSH-Px (*p* < 0.01), SOD (*p* < 0.01) and T-AOC(*p* < 0.01) activities were significantly decreased in the serum of the model group compared to the sham group (Figures 4A,C,D). N2FBR increased GSH-Px (*p* < 0.01), SOD (*p* < 0.01) and T-AOC (*p* < 0.01) levels in comparison with the model group. Similarly, those antioxidant enzymes levels were increased in the SB group. Moreover, the serum MDA level, an index of lipid peroxidation, had been significantly increased in the model group than in the sham group (*p* < 0.01). BLM-mediated lipid peroxidation was significantly decreased following the administration of N2FBR and SB (Figure 4B) (*p* < 0.01). These data suggest that N2FBR is involved in Bleomycin-induced oxidative stress regulation.

### Number 2 Feibi Recipe Promotes Autophagy in Bleomycin-Induced Pulmonary Fibrosis Mice

**FIGURE 5:**
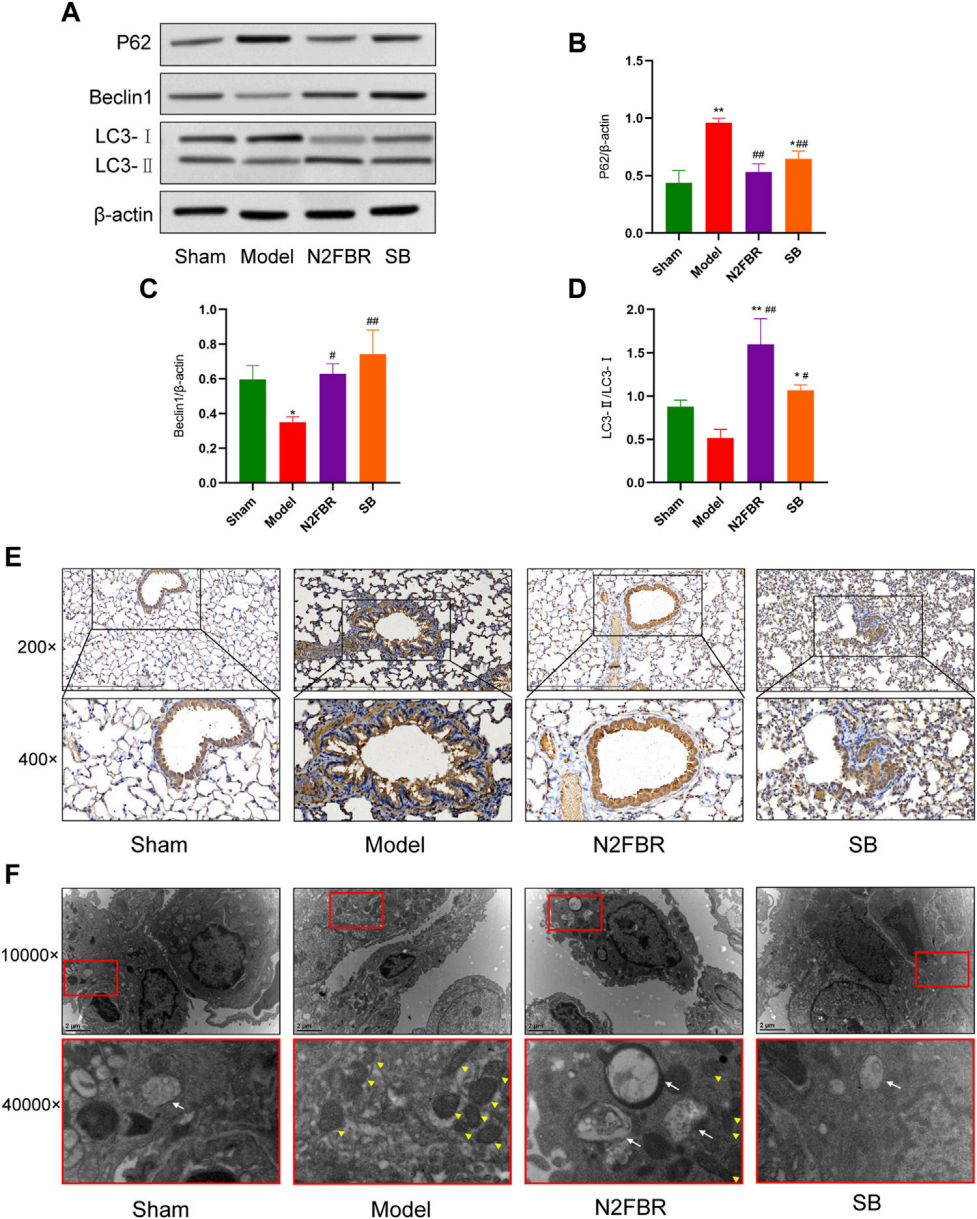
Effects of N2FBR on autophagy. **(A)** Protein levels of beclin-1, p62, and LC3II as measured by western blotting. **(B–D)** Relative density values showing P62, beclin1, LC3- II/LC3- I expression. **(E,F)** Representative images of TEM and immunohistochemical staining for P62. The white arrows indicate autophagosomes and autolysosomes, the yellow arrowheads indicate abnormal mitochondria. Data are expressed as *p < 0.05, **p < 0.01, compared with sham group; #*p* < 0.05, ##*p* < 0.01, compared with model group.

The expression of important autophagy-related proteins, LC3-I, LC3-II, p62, and Beclin1 was measured to confirm the N2FBR is regulated by autophagy (Figure 5A). p62 is a cargo receptor for autophagy. Its accumulation levels are proportional to the degree of autophagic impairment (Jiang and Mizushima, 2015). Compared to the sham group, the expression level of P62 in the model group was significantly enhanced (*p* < 0.01). P62 protein expression was significantly decreased following treatment with N2FBR (*p* < 0.01) and SB (*p* < 0.01) (Figure 5B). The findings had been confirmed by immunohistochemistry of P62 (Figure 5E). The model group had significantly lower level of beclin1 protein expression than the sham group (*p* < 0.05). Treatment with N2FBR (*p* < 0.05) and SB (*p* < 0.01) increased the level of expression of beclin1 (Figure 5C). The ratio of LC3-II/LC3-Ⅰ is widely utilised as an indicator of autophagy. N2FBR treatment significantly accelerated the conversion of LC3B-I to LC3B-II. However, no discernible difference had been observed between the sham and model groups (Figure 5D). For detecting autophagic flux, TEM is the gold standard. In this study, TEM had been used for observing the morphological changes of the lung tissue. As shown in Figure 5F, cells from the model group exhibited few autophagic vesicles, while numerous mitochondria were swollen, enlarged, and the cristae distorted. The use of N2FBR and SB increased the production of autophagic vesicles and reduced the number of abnormal mitochondria. Taken together, these findings proposed that N2FBR promotes autophagy, which might be responsible for its effects in the alleviation of pulmonary fibrosis.

### Number 2 Feibi Recipe Relieves Lysosomal Damage in Bleomycin-Induced Pulmonary Fibrosis Mice

**FIGURE 6:**
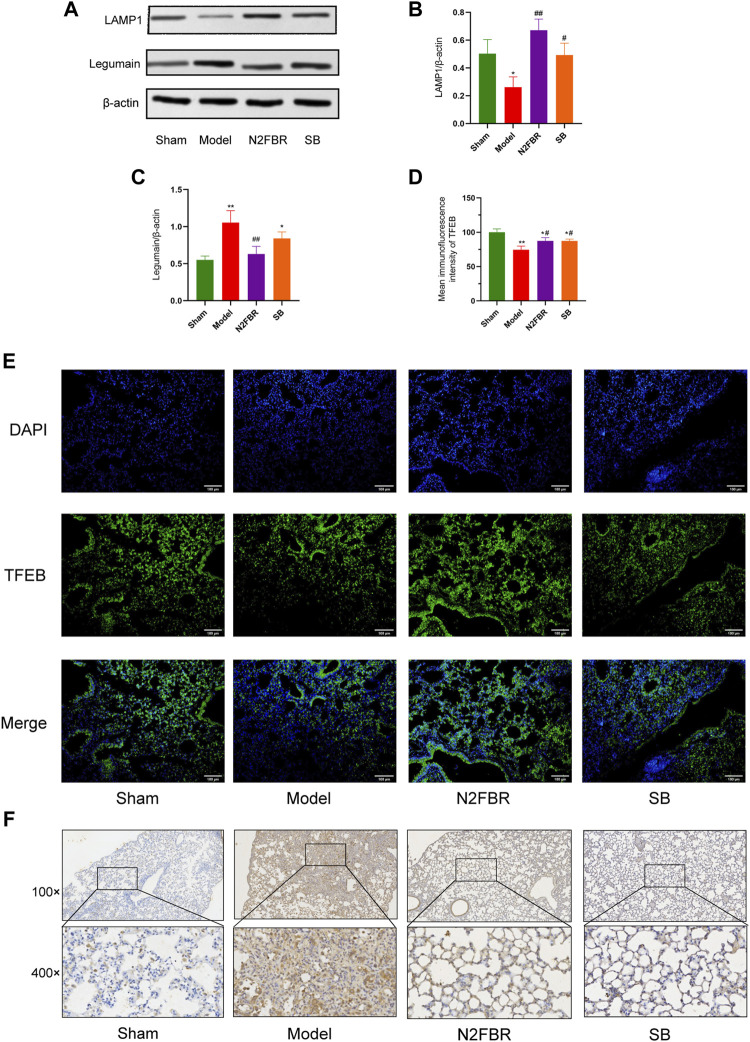
Effects of N2FBR on lysosome injury. **(A)** Protein levels of LAMP1 and Legumain as determined by western blotting. Relative density values showing **(B)** LAMP1 and **(C)** Legumain expression. **(D)** Mean immunofluorescence intensity of TFEB. **(E)** Representative images of immunofluorescence showing the expression of TFEB. **(F)** Representative images of immunohistochemical staining for Legumain. Data are expressed as *p < 0.05, **p < 0.01, compared with sham group; #*p* < 0.05, ##*p* < 0.01, compared with model group.

The expression LAMP1, Legumain, and TFEB was detected. In contrast to the sham group, the expression level of LAMP1 in the model group was inhibited (Figure 6A) (*p* < 0.05), while the expression in both the N2FBR (*p* < 0.01) and SB (*p* < 0.05) groups was significantly upregulated (Figure 6B). As demonstrated in Figure 6C, the expression level of Legumain in the model group had been significantly enhanced (*p* < 0.01). Legumain protein expression was significantly decreased following treatment with N2FBR (*p* < 0.01). Nevertheless, the level of Legumain in the SB group was not crucially different from that of the model group. Immunohistochemistry of legumain validated the findings (Figure 6F). Immunofluorescence confirmed that nuclear translocation and expression of TFEB was inhibited in BLM-induced mice (Figures 6D,E), and this phenomenon was reversed by N2FBR (*p* < 0.05) and SB (*p* < 0.05) treatment. It is implied by these findings that BLM-induced pulmonary fibrosis is caused by lysosome disorders. N2FBR alleviates BLM-induced lysosomal damage.

### Number 2 Feibi Recipe Activates the GSK-3β/mTOR Signalling Pathway to Trigger Autophagy

**FIGURE 7:**
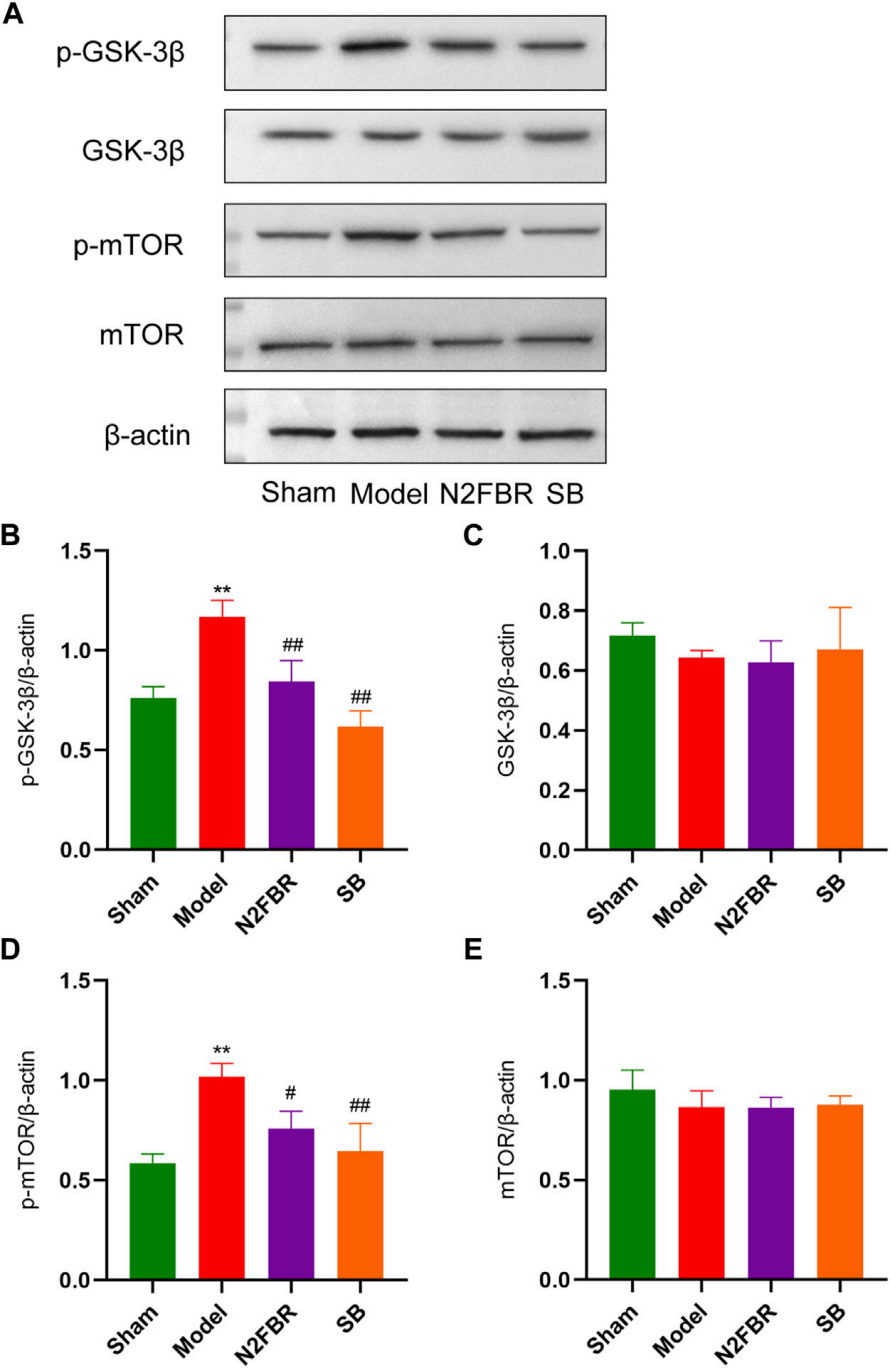
N2FBR activated the GSK-3β/mTOR signaling pathway. **(A)** Protein levels of p-GSK-3β, GSK-3β, p-mTOR, and mTOR as quantified by western blotting. Relative density values showing **(B)** p-GSK-3β, **(C)** GSK-3β, **(D)** p-mTOR and **(E)** mTOR expression. Data are expressed as **p < 0.01, compared with sham group; #*p* < 0.05, ##*p* < 0.01, compared with model group.

Western blotting was used to determine the levels of GSK-3β and mTOR, and their phosphorylation, which is the autophagy regulation mechanism, to see how N2FBR activated autophagy. As demonstrated in Figure 7A, the model group showed significantly increased expression levels of phosphorylated mTOR (p-mTOR) (*p* < 0.01) and phosphorylated GSK-3β(p-GSK-3β) (*p* < 0.01) in contrast to the sham group. After N2FBR (*p* < 0.05, *p* < 0.01) and SB (*p* < 0.01, *p* < 0.01) treatment, the levels of p-mTOR and p-GSK-3β were decreased (Figures 7B,D). Nevertheless, no discernible difference in total GSK-3β and total mTOR protein expression was observed in each group (Figures 7C,E). These findings indicated that N2FBR could inhibit the GSK-3β/mTOR pathway in BLM-induced pulmonary fibrosis mice.

## Discussion

IPF is a chronic, persistent pulmonary disease caused by a variety of factors. Unfortunately, there are not many options in terms of available pharmacological therapies. In China, Number 2 Feibi Recipe was used in clinic for the treatment of pulmonary fibrosis. Our previous study confirmed that the N2FBR decrease oxidative damage in mice exposed to PM2.5 (Liu et al., 2018). Furthermore, N2FBR reduced the level of intracellular ROS and regulated the balance of autophagy and apoptosis in H_2_O_2_-mediated AECs (Gu et al., 2022). In the current research, we demonstrated that N2FBR had anti-fibrotic therapeutic effects in BLM-induced pulmonary fibrosis and further explored the effector mechanisms.

In rodent models of pulmonary fibrosis, bleomycin intratracheal instillation is commonly employed. In BLM-induced mice, ECM deposition in enhanced, resulting in alveolar wall thickening and reduced ventilation function. Fibrosis could be histologically detected by day 14, with obvious responses which occur between days 21–28 (Moore and Hogaboam, 2008). Col-I and Col-III are the main components of extracellular matrix. α-SMA is a commonly recognized indicator for pulmonary fibrosis. Reduced α-SMA expression reduces fibroblasts activation, resulting in a less excessive ECM deposition (Liu T. et al., 2021). Hydroxyproline accounts for 12.5% of the amino acid content in collagen fibers. Hydroxyproline expression might be linked to the level of ECM. TGF-β1 was described as a critical signalling molecule engaged in fibrosis-induced lung injury and is considered as a key modulator of pulmonary fibrosis. TGF-β1 could activate downstream proteins and eventually initiate the induction of numerous fibrosis-related proteins (Fernandez and Eickelberg, 2012). Our findings showed that N2FBR improved the histological properties of lung tissues and slowed the advancement of fibrosis in this investigation. Moreover, N2FBR inhibited the increase in α-SMA, collagen I, collagen III, hydroxyproline, and TGF-β1 induced by bleomycin. These results comply with our previous hypotheses and further supported the anti-fibrotic effects of N2FBR. It may be a promising new agent for the treatment of pulmonary fibrosis.

Lung injury and pulmonary fibrosis are known to be linked to oxidate stress. At low concentrations, ROS could modulate signal transduction. Nevertheless, ROS at high concentrations could cause damage to lipids, proteins, and nucleic acids, resulting in cell death and tissue damage. Lung tissue as well as biological fluids from IPF patients illustrated that oxidative stress significantly contributes to IPF development and progression (Landi et al., 2014). TGF-β, the most potent pro-fibrotic factor, has been implicated in the production of reactive oxygen species (ROS), which has activated fibrogenic factors. As a result, a vicious circle is formed (Wang et al., 2022). The lung possesses an endogenous antioxidant system which protects against oxidative stress damage. In order to remove ROS, various anti-oxidant molecules and enzymes could be produced. In the present study, MDA, SOD, T-AOC, GSH-Px were selected as markers to evaluate bleomycin-induced oxidative stress damage. MDA, a lipid peroxidation product, has been utilised as an indicator of the cell damage caused by oxidative stress (Sun et al., 2020). SOD and GSH-Px are free radical scavengers that are capable of removing the active substances in oxidative damage (Poprac et al., 2017). The T-AOC is an index that measures the antioxidant capacity of the body. MDA levels were dramatically elevated after bleomycin treatment, while SOD, GSH-Px, and T-AOC levels were reduced (Dong et al., 2017). MDA expression level was decreased, while SOD, GSH-Px, T-AOC had been increased in the N2FBR group in comparison with model group. In a nutshell, N2FBR protects against oxidative damage by enhancing the activity of antioxidant enzymes in pulmonary fibrosis.

Autophagy is a homeostatic degradation process that aids in the elimination of unfolded proteins or damaged organelles and regulates cell metabolism through autophagy-related genes (Zhao et al., 2020). Beclin-1, an autophagy-related protein, has been engaged in the activation of autophagy as well as the development of autophagosome precursors. Microtubule-associated proteins 1A/1B light chain 3 (LC3) is a marker of autophagy. In the early stages of autophagy formation, LC3B-I has been converted into LC3B-II (Nieto-Torres et al., 2021). The degree of autophagy relies on the LC3II/I ratio. P62 takes part in the maturation of the autophagosome and has also been degraded during autophagy. Autophagy impairment can be confirmed by p62 accumulation. Previous research has found that the expression levels of LC3-II had been reduced in lung tissue of patients with IPF (Ricci et al., 2013). The inhibition of autophagy enhances epithelial cell senescence and myofibroblast differentiation in lung fibrosis (Araya et al., 2013). Epithelial-mesenchymal transition (EMT) is aided by abnormal autophagy in alveolar epithelial cells (Hill et al., 2019). It has been revealed by our previous research that N2FBR alleviated H_2_O_2_-mediated oxidative stress damage in AECs via promoting autophagy (Gu et al., 2022). In this study, we analyzed the expression of LC3-II/LC3-I, beclin1, and p62 in mice lung tissues. N2FBR administration resulted in a considerable down-regulation of p62, whereas the beclin-1 expression and LC3-I to LC3-II transformations were simultaneously stimulates as predicted. TEM is commonly recognised as the gold standard for the qualitative detection of autophagy. As apparent in the TEM images, numerous dysmorphic and enlarged mitochondria, and a few autophagic vesicles were observed in lung tissues of the model group. An increasing number of autophagic vesicles were found after N2FBR therapy. The number of abnormal mitochondria was decreased in N2FBR group compared with the model group. It is evident from these results that the functions of N2FBR on upregulating the expression of autophagy-related proteins and enhancing autophagosome formation.

Lysosomes are acid organelles for degradation at the end of the autophagic flux. The fusion of autophagosome and lysosome is the rate-limiting step of autophagy (El-Kadi et al., 2012). Excess ROS induce lysosomal permeabilization and destabilization, which lead to the disruption of autophagy-lysosome pathway. Lysosomal Associated Membrane Protein-1 (LAMP1), a major protein component of the lysosomal membrane (Eskelinen, 2006), is already established as a general marker of lysosome dysfunction. Legumain is mainly localized to the acidic lysosomal compartments. Lysosome protease is released into intracellular regions when lysosomal membranes are damaged (Akhtar et al., 2015). TFEB is a master transcriptional regulator of autophagy as well as lysosome biogenesis. Activation of TFEB is essential for lysosomal homeostasis after lysosomal damage (Nakamura et al., 2021). Silica inhibited autophagy flux via interfering with lysosomal degradation. Overexpression of the transcription factor TFEB reduced lysosomal dysfunction and silica-induced lung fibrosis (He et al., 2020). In the current study, bleomycin treatment crucially downregulated LAMP1 expression and upregulated Legumain expression, which had been reversed by N2FBR treatment. Immunofluorescence demonstrated that N2FBR treatment promoted nuclear translocation and expression of TFEB. It is suggested by these findings that the lysosome is injured by BLM, and N2FBR treatment might protect lysosomal function as well as promote autophagic substrate degradation. Nevertheless, a recent study shown that azithromycin reduced collagen production in TGF-β treated fibroblasts by disrupting lysosomal function (Krempaska et al., 2020). These discrepancies could be related to the use of different experimental models of pulmonary fibrosis. Lysosome dysfunction might play varying roles in distinctive cells involved in IPF. Considering that little attention has been paid to this area, additional research should be conducted in future studies.

GSK-3β is involved in the control of a variety of downstream signalling pathways. SB216763, a GSK-3 inhibitor, has previously been shown to protect against BLM-induced pulmonary fibrosis. In pulmonary fibroblasts, silencing GSK-3β with siRNA decreased the TGF-β1-induced expression of α-actin and fibronectin (Baarsma et al., 2013). Additionally, the knockdown of GSK-3β reversed the induction of EMT in alveolar epithelial cells (Liu et al., 2021). GSK-3β serves a pivotal role in the regulation of mTOR pathway. The basal levels of mTORC1 and mTORC2, as well as the mTOR complexes, were shown to be up-regulated in IPF lung samples (Chang et al., 2014; Romero et al., 2016). Inhibiting mTOR accelerated autophagy, reversing apoptotic resistance in fibroblasts from IPF patients. In lung epithelial cells, Rapamycin inhibition reduced mitochondrial oxidative damage and delayed cellular senescence (Summer et al., 2019). The protein expression level of p-GSK-3β, p-mTOR, and the total of GSK-3β and mTOR in the lungs of mice were determined by Western blotting in the current study. We explored that N2FBR inhibited the activation of p-GSK-3β and p-mTOR, but had no effect on total GSK-3β and mTOR, which illustrates that the beneficial effect of N2FBR may be attributed to the inhibition of the GSK-3β/mTOR pathway.

In conclusion, the current investigation confirmed N2FBR as a promising therapy candidate for idiopathic pulmonary fibrosis collagen deposition and oxidative stress. Additionally, it activated autophagy and protected the lysosome from injury. Mechanistically, these functions may be associated with the regulation of the GSK-3β/mTOR pathway. These findings provide experimental evidence that N2FBR exert its function through promotion of autophagy and offering a new therapeutic strategy for the prevention of IPF. Nevertheless, additional research has been required to confirm whether N2FBR alleviates pulmonary fibrosis by modulating autophagy.

## Data Availability

The raw data supporting the conclusions of this article will be made available by the authors, without undue reservation.
